# Study on the Role of FHL2 in Chicken: Promoting Bone Growth and Inhibiting Fat Formation via Regulating BMSC Differentiation Balance

**DOI:** 10.3390/ani16203197

**Published:** 2026-10-12

**Authors:** Zijian Qin, Lisha Han, Fen Ying, Ming Pan, Changbin Zhao, Tao Lin, Shuibing Liu, Linxi Wu, Bolin Cai, Qingbin Luo, Xiquan Zhang, Dexiang Zhang, Farhad Bordbar, Hongmei Li

**Affiliations:** 1 Science and Guangdong Laboratory for Lingnan Modern Agriculture, South China Agricultural University, Guangzhou 510642, China; 20243139808@stu.scau.edu.cn (Z.Q.); 20242024008@stu.scau.edu.cn (L.H.); 18271426970@163.com (F.Y.); 17311143943@163.com (M.P.); zcbscau@stu.scau.edu.cn (C.Z.); 13423781398@163.com (T.L.); 20241024012@stu.scau.edu.cn (S.L.); 15816038118@163.com (L.W.); bolincai@scau.edu.cn (B.C.); qbluo@scau.edu.cn (Q.L.); xqzhang@scau.edu.cn (X.Z.); zhangdexiang0001@sina.com (D.Z.); 2 Guangdong Provincial Key Lab of Agro-Animal Genomics and Molecular Breeding and Key Lab of Chicken Genetics, Breeding and Reproduction, Ministry of Agriculture, Guangzhou 510642, China; 3 State Key Laboratory for Conservation and Utilization of Subtropical Agro-Bioresources, South China Agricultural University, Guangzhou 510642, China

**Keywords:** bone formation, fat deposition, stem cell differentiation, apoptosis, transcriptome sequencing analysis

## Abstract

In this study, the objective was to identify the key regulators of the osteogenic–adipogenic differentiation equilibrium of chicken bone marrow mesenchymal stem cells (BMSCs), with the expectation of developing marker genes that promote skeletal development and inhibit excessive fat deposition. BMSCs were isolated and phenotypically characterized. qRT-PCR showed *FHL2* increased during osteogenic differentiation. Transfection with *FHL2* overexpression plasmid or siRNAs was done. *FHL2* overexpression increased osteogenic marker mRNA (*RUNX2*, *ALP*, *Osterix*, and *OCN*) levels, reduced lipid deposition-related gene (*C/EBPα*, *C/EBPβ*) expression, and increased mineralized area and nodules. *FHL2* knockdown had the opposite effect. *FHL2* overexpression also reduced apoptosis and proliferation-related transcripts, decreased apoptosis rate, and reduced proliferation-related transcripts and altered cell-cycle distribution. After *FHL2* knockdown, RNA sequencing identified 81 differentially expressed genes. Kyoto Encyclopedia of Genes and Genomes (KEGG) enrichment showed changes in various processes and pathways. Enrichment analysis indicated that the differentially expressed genes were involved in apoptosis, cytokine–cytokine receptor interaction, Th17 cell differentiation, JAK-STAT signaling, cAMP signaling, steroid hormone biosynthesis, and retinol metabolism. These findings indicate that FHL2 promotes osteogenic differentiation and suppresses adipogenic differentiation in chicken BMSCs in vitro and may act through multiple downstream pathways.

## Simple Summary

In some chickens, high body weight is accompanied by a low dressing percentage because growth is preferentially directed to bone and fat rather than muscle. Our study identified four and a half LIM domains protein 2 (*FHL2*) as a key gene that promoted osteogenic differentiation and suppressed adipogenic differentiation in bone marrow stem cells. This finding supports the future development of molecular markers for selecting chickens with better carcass yield and healthier body composition. In the long term, improving the bone–lipid balance may increase production efficiency, reduce feeding costs, and lower the risk of skeletal problems.

## 1. Introduction

In recent years, chicken has become the second most consumed meat in China after pork and contributes more than half of the country’s poultry meat production [1]. Although poultry meat production has increased rapidly, consumer demand for high-quality poultry meat has also continued to rise. Breeding strategies that focus mainly on rapid growth can neglect balanced development. As a result, broilers may develop larger skeletal frames, accumulate more abdominal fat, exhibit lower dressing percentage, and even exhibit muscle degeneration. These outcomes impose substantial health and economic burdens on the poultry industry [2]. Therefore, chicken breeding programs should aim to maintain coordinated development of bone, adipose tissue, and skeletal muscle. Identifying the genetic regulators of the balance between osteogenic and adipogenic differentiation will help reduce skeletal diseases and improve carcass quality. It may also increase economic returns and provide a molecular basis for selecting superior poultry lines. These advances may support sustainable genetic improvement in the poultry industry.

FHL2 contains four and a half LIM domains and is a member of the LIM-domains protein family. Through its LIM domains, FHL2 can interact with more than 50 distinct proteins, including transcription factors, integrins, and cytoskeletal proteins [3]. FHL2 functions as a multifunctional scaffold and transcriptional cofactor by interacting with multiple proteins, including androgen receptor, IGFBP-5 and CREB [4,5,6]. FHL2 is also involved in mechanotransduction and can regulate cellular processes such as proliferation and differentiation [7]. Additionally, FHL2 can interact with multiple key regulators of bone formation. Recent studies indicate that FHL2 promotes osteogenic differentiation through β-catenin-related mechanisms and may attenuate inflammatory bone damage, whereas androgen receptor signaling contributes to bone formation and fracture repair in skeletal progenitor cells [8,9,10]. IGFBP-5 contains a conserved nuclear localization sequence and can be imported into the nucleus through an importin-α/importin-β-dependent pathway. Recent reviews also note that IGFBP-5 has been reported to interact with FHL2 and to modulate IGF signaling in bone cells through extracellular-matrix-related mechanisms [5,11]. These studies indicated that FHL2 can function as a signal transduction mediator. In addition, FHL2 can interact with RUNX2, a key regulator of osteoblast function. Studies have reported that, in cultured human periodontal ligament cells in vitro, FHL2 enhances the transcriptional activity of RUNX2 and may contribute to the mineralization process in these cells [9]. Notably, FHL2 can interact with β-catenin, and the interaction may be either positive or negative depending on the cellular context. In contrast to its inhibitory effect on β-catenin/TCF signalling in myoblasts [12], FHL2 promotes β-catenin nuclear translocation and serves as a coregulator for β-catenin-mediated transcription in renal tubular epithelial cells [13]. Previous studies have reported that four and a half LIM domain protein 2 (FHL2) promotes osteoblastic differentiation of mouse and human mesenchymal stem cells (MSCs) by facilitating β-catenin nuclear enrichment and Wnt/β-catenin signaling-dependent RUNX2 expression [9]. However, the role of FHL2 in the osteogenic and adipogenic differentiation of chicken mesenchymal stem cells remains unclear. p53 is an important tumor suppressor protein that can induce apoptosis and senescence under different conditions and plays a crucial role in cell-cycle regulation and the maintenance of genomic integrity [14]. FHL2 has also been implicated in p53-related signaling during the osteogenic differentiation of mesenchymal stem cells [9]. In addition, FHL2 has been implicated in the onset and progression of non-neoplastic disorders, such as ovarian cysts and diabetes. *FHL2* knockout can impair follicular development and fertility by attenuating EGF/EGFR/YAP signaling in ovarian granulosa cells [15]. In FHL2-deficient mice, glucose-stimulated insulin secretion is improved, whereas elevated *FHL2* expression in humans has been associated with type 2 diabetes [16].

As a scaffold protein, FHL2 regulates cellular signal transduction and gene transcription and plays an important role in processes such as cell-cycle control, proliferation, and differentiation. These findings have been demonstrated primarily in humans and mice. However, the regulatory mechanisms of FHL2 during skeletal growth and development in chickens have rarely been reported. Given its important role in osteogenic differentiation, FHL2 needs further investigation.

Previous studies reported that high *FHL2* expression promotes osteogenic differentiation, affects adipogenic differentiation, and inhibits myogenic differentiation. These effects are likely related to the unique functional properties of FHL2. In chickens, bone is formed primarily through the differentiation of bone marrow-derived mesenchymal stem cells (BMSCs) into osteoblasts, followed by matrix deposition and mineralization [17]. Although MSCs derived from distinct tissues exhibit differences in differentiation capacity and transcriptional profiles, they retain the ability to complete osteogenic and adipogenic differentiation under defined in vitro induction conditions. In broiler chickens, compact-bone-derived MSCs have been shown to adhere to plastic, form colonies, and undergo osteogenic, adipogenic, and myogenic differentiation, supporting chicken MSCs as a useful model for studying lineage allocation in vitro [18].

Based on our preliminary findings, this study further investigated the specific effects of *FHL2* overexpression and knockdown on the osteogenic–adipogenic balance in chicken bone marrow mesenchymal stem cells. These findings provide new insight into the molecular regulation of bone–lipid balance in chickens and support the future development of molecular markers for poultry genetic improvement.

## 2. Materials and Methods

### 2.1. Study Design

Chicken bone marrow mesenchymal stem cells (BMSCs) were isolated from 8-day-old male and female Yangshan chickens and expanded in vitro. No other chicken breeds were included. Unless otherwise stated, experiments were performed using three independent biological replicates per group. Biological replicates were generated from independent donor chicken, with BMSCs isolated and cultured separately from each donor rather than repeated measurements from a single pooled cell batch. Passage-3 BMSCs were used for osteogenic and adipogenic induction, *FHL2* overexpression or knockdown, and downstream assays including qRT-PCR, histochemical staining, and flow-cytometric analyses.

### 2.2. Ethical Statement on Animal Experiments

All animal procedures were approved by the Animal Welfare Ethics Review Committee of South China Agricultural University and conducted in accordance with the relevant standards for animal welfare (approval ID: SCAU#0017).

### 2.3. Isolation and Culture of Chicken BMSCs

Chickens were disinfected with 75% ethanol (MacLean, Guangzhou, China), and all procedures were performed under sterile conditions. Bone marrow was flushed to obtain a cell suspension, filtered through a 70 μm cell strainer, and centrifuged at 500× *g* for 5 min (Eppendorf, Hamburg, Germany). The pellet was washed twice (250× *g*, 10 min) and resuspended in complete DMEM/F-12 (Gibco, Grand Island, NY, USA) medium supplemented with fetal bovine serum (Gibco, Grand Island, NY, USA) and penicillin–streptomycin (Solarbio, Beijing, China). Cells were seeded and incubated at 37 °C in 5% CO_2_. After 24 h, the medium was replaced to remove non-adherent cells. Adherent cells were washed with PBS and maintained with medium changes every 2–3 days. Cells were passaged at >85% confluence and used at passage 3.

### 2.4. Phenotypic Characterization of Chicken BMSCs by Immunofluorescence

Passage-3 chicken bone marrow-derived cells were fixed with 4% paraformaldehyde (Biosharp, Hefei, China) and subjected to immunofluorescence staining for the mesenchymal-associated markers CD29, CD105, and CD166, as well as the hematopoietic-associated marker CD45 (Invitrogen, Frederick, MD, USA). After incubation with the primary antibodies and FITC-conjugated secondary antibodies, fluorescence images were acquired using an inverted fluorescence microscope (Lecia, Wetzlar, Germany). The use of these or comparable antibodies for the characterization of chicken-derived bone marrow/stem-cell populations has been reported previously [19].

### 2.5. Osteogenic and Adipogenic Induction and Histochemical Staining

Passage-3 BMSCs were seeded in 6-well plates (2 mL per well) and cultured to ~80% confluence. The cells used for differentiation were obtained from independent batches that were separately expanded before induction. Two-thirds of the complete medium was replaced with an equal volume of osteogenic or adipogenic induction medium. After 2 days, two-thirds of the induction medium was replaced, and the medium was subsequently renewed every 2 days. Morphological changes were monitored up to day 7. Cells were rinsed twice with PBS (Solarbio, Beijing, China), fixed with 4% paraformaldehyde for 25 min at room temperature, and washed three times with PBS. Osteogenic differentiation was evaluated by Alizarin Red S staining (30 min, room temperature) (Solarbio, Beijing, China), and adipogenic differentiation was evaluated by Oil Red O staining (60 min, room temperature) (Solarbio, Beijing, China). Images were recorded using an inverted microscope (Lecia, Shanghai, China).

### 2.6. Primer Design, RNA Extraction, Reverse Transcription, and qRT-PCR

Primers were designed in part using Primer-BLAST (NCBI, Bethesda, MD, USA; Primer3 v2.5.0) and synthesized commercially. Total RNA was extracted using Trizol (Takara, Shiga, Janpan) following the manufacturer’s instructions. Reverse transcription was performed using a commercial kit to generate cDNA. Quantitative real-time PCR was performed using SYBR-based chemistry (Vazyme, Nanjing, China)on a real-time PCR system. For qRT-PCR, three independent biological replicates were analyzed for each group. Each biological replicate was analyzed with 3–4 technical replicates, and the technical replicate values were averaged before statistical analysis. Relative expression was calculated using the 2^−ΔΔCt^ method. Gene expression levels were normalized to *GAPDH* as the internal reference, and primer sequences are provided in Supplementary Table S1.

### 2.7. Cell Transfection

The *FHL2* overexpression plasmid was purchased from Wuhan Genecreat Co., Ltd. (Wuhan, China). Endotoxin-free plasmid DNA was prepared according to the manufacturer’s instructions, quantified spectrophotometrically, and stored at −20 °C. The *FHL2* siRNA was designed based on the chicken *FHL2* mRNA sequence retrieved from NCBI and acquired from Ribobio Biotechnology Co., Ltd. (Guangzhou, China). BMSCs were seeded in 12-well plates and transfected using Lipofectamine^®^ 3000 (Thermo Fisher Scientific, Waltham, MA, USA) according to the manufacturer’s protocol. Overexpression transfection was performed at 70% confluence, and knockdown transfection was performed at 50% confluence. Cells were collected at the indicated time points for subsequent analyses. Information about siRNA and plasmid synthesis is in Supplementary Material File S2.

### 2.8. Cell-Cycle and Apoptosis Analysis

For flow-cytometric analyses, log-phase BMSCs were seeded in 6-well plates and transfected for *FHL2* overexpression or knockdown. Cells were collected 48 h post-transfection, washed with PBS, and processed according to the instructions of the cell-cycle and apoptosis detection kits. For ethanol fixation, ice-cold absolute ethanol was added dropwise while vortexing, and cells were fixed at 4 °C for 30 min (or stored at −20 °C). Samples were analyzed on a CytoFLEX flow cytometer, and data were processed using FlowJo software version 10.10.2 (BD Life Sciences, Ashland, OR, USA).

### 2.9. RNA Sequencing and Bioinformatic Analysis

Total RNA was extracted from chicken BMSCs transfected with si-*FHL2* or si-NC, with three biological replicates per group. RNA concentration and purity were assessed using a NanoDrop 2000 spectrophotometer (Thermo Fisher Scientific, Waltham, MA, USA), and RNA integrity was evaluated using an Agilent 2100 Bioanalyzer (Agilent Technologies, Santa Clara, CA, USA). Qualified RNA samples were used for cDNA library construction and sequenced on an Illumina NovaSeq 6000 platform (Illumina, San Diego, CA, USA) to generate paired-end 150-bp reads.

Raw reads were filtered to remove adaptor sequences and low-quality reads. The resulting clean reads were mapped to the chicken reference genome GRCg6a using HISAT2. Transcript assembly and gene expression quantification were performed using StringTie. Gene-level raw read counts, rather than FPKM or TPM values, were used as input for the limma-voom analysis. Differential expression analysis between the si-FHL2 and si-NC groups was performed using the limma-voom pipeline in R. Genes with |log2(fold change)| ≥ 1 and an unadjusted *p* value < 0.05 were considered differentially expressed. GO and KEGG enrichment analyses were performed for the identified differentially expressed genes, and terms with adjusted *p* < 0.05 were considered significantly enriched.

### 2.10. Statistical Analysis

Data are expressed as the mean ± standard error of the mean (SEM). Data normality and homogeneity of variances were assessed using the Shapiro–Wilk test and Levene’s test in SPSS v26.0, respectively, where applicable. Differences between two groups were analyzed using a *t*-test. For comparisons among multiple groups, one-way analysis of variance (one-way ANOVA) was performed in SPSS v26.0. The analysis results were analyzed via GraphPad Prism 8.0 (GraphPad Software, Inc., San Diego, CA, USA). Statistical significance was defined as *p* < 0.05, with significance levels denoted as * *p* < 0.05, ** *p* < 0.01, *** *p* < 0.001, and **** *p* < 0.0001. Different letters indicate significant differences among groups (*p* < 0.05).

## 3. Results

### 3.1. Isolation and Phenotypic Characterization of Chicken BMSCs

**Figure 1:**
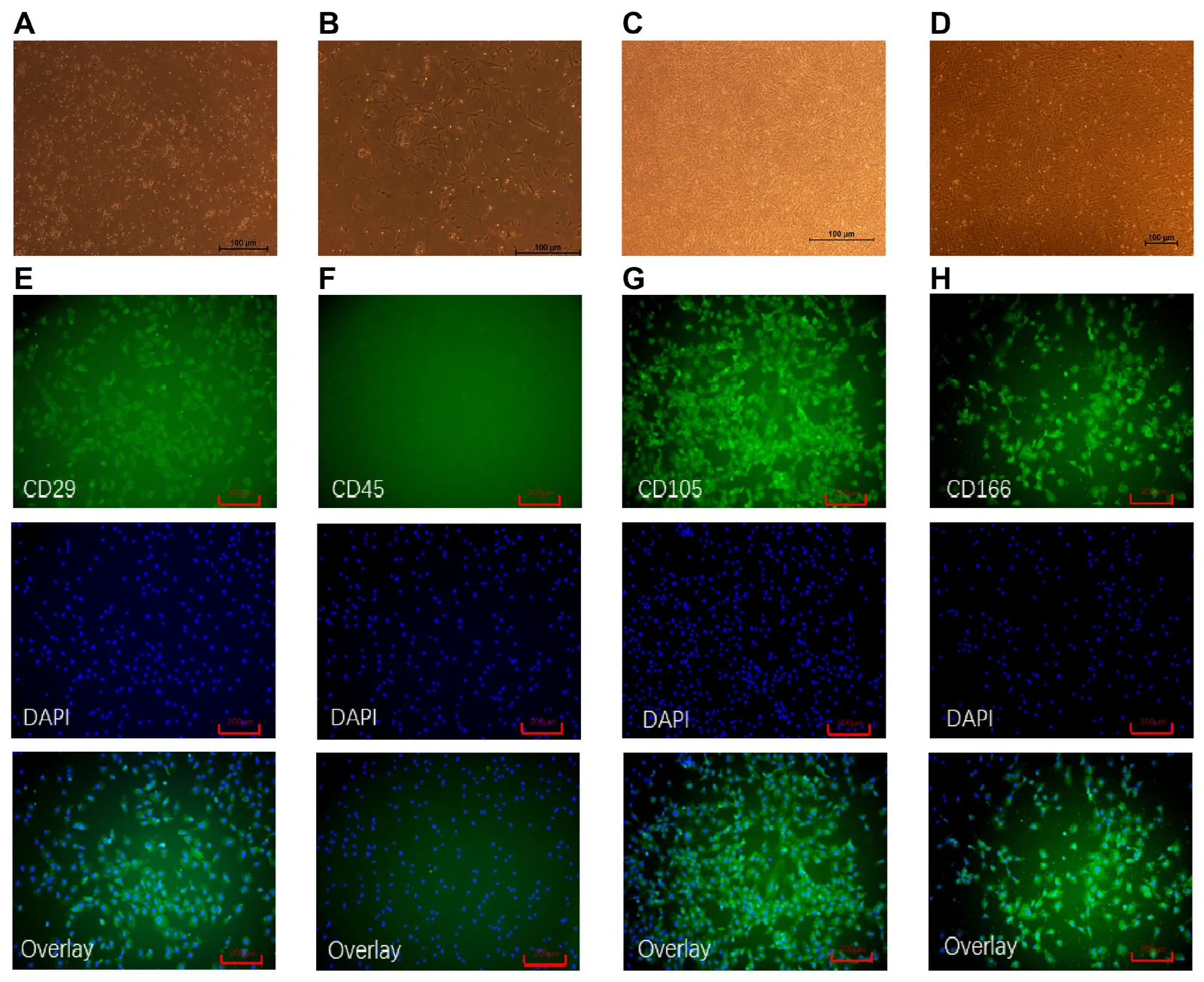
Isolation and immunofluorescence characterization of chicken bone marrow-derived cells. (**A**–**D**) Representative phase-contrast images showing cell morphology at 24 h (**A**), day 3 (**B**), day 9 (**C**), and 24 h after passaging (**D**). Scale bars = 100 μm. (**E**–**H**) Immunofluorescence staining of passage-3 cells for CD29 (**E**), CD45 (**F**), CD105 (**G**), and CD166 (**H**). Nuclei were counterstained with DAPI, and merged images are shown as overlays. Scale bars = 200 μm.

Primary cells isolated from the bone marrow of 8-day-old Yangshan chickens initially appeared as heterogeneous, oval-shaped cells in suspension. After 24 h, cells began to adhere, remained sparsely distributed, and exhibited an irregular polygonal morphology; medium replacement effectively removed non-adherent cells (Figure 1A). By day 3, adherent cells were predominantly spindle-shaped with variable lengths (Figure 1B). By day 9, cultures reached approximately 80% confluence and were passaged; cells displayed a more uniform fibroblast-like morphology after passaging, and passage-3 cells were used for subsequent experiments (Figure 1C,D). Immunofluorescence staining of passage-3 chicken bone marrow-derived cells showed positive signals for CD29, CD105, and CD166, whereas no detectable signal for the hematopoietic-associated marker CD45 was observed (Figure 1E–H). Together with their adherent, fibroblast-like morphology and their capacity for osteogenic and adipogenic differentiation, these findings support the characterization of the isolated cells as chicken bone marrow-derived stromal/MSC-like cells. However, immunofluorescence staining alone does not establish a highly purified MSC population. Accordingly, the cells are referred to as chicken BMSCs hereafter for consistency with the experimental design.

### 3.2. Osteogenic Differentiation of Chicken BMSCs and Upregulation of FHL2 During Induction

**Figure 2:**
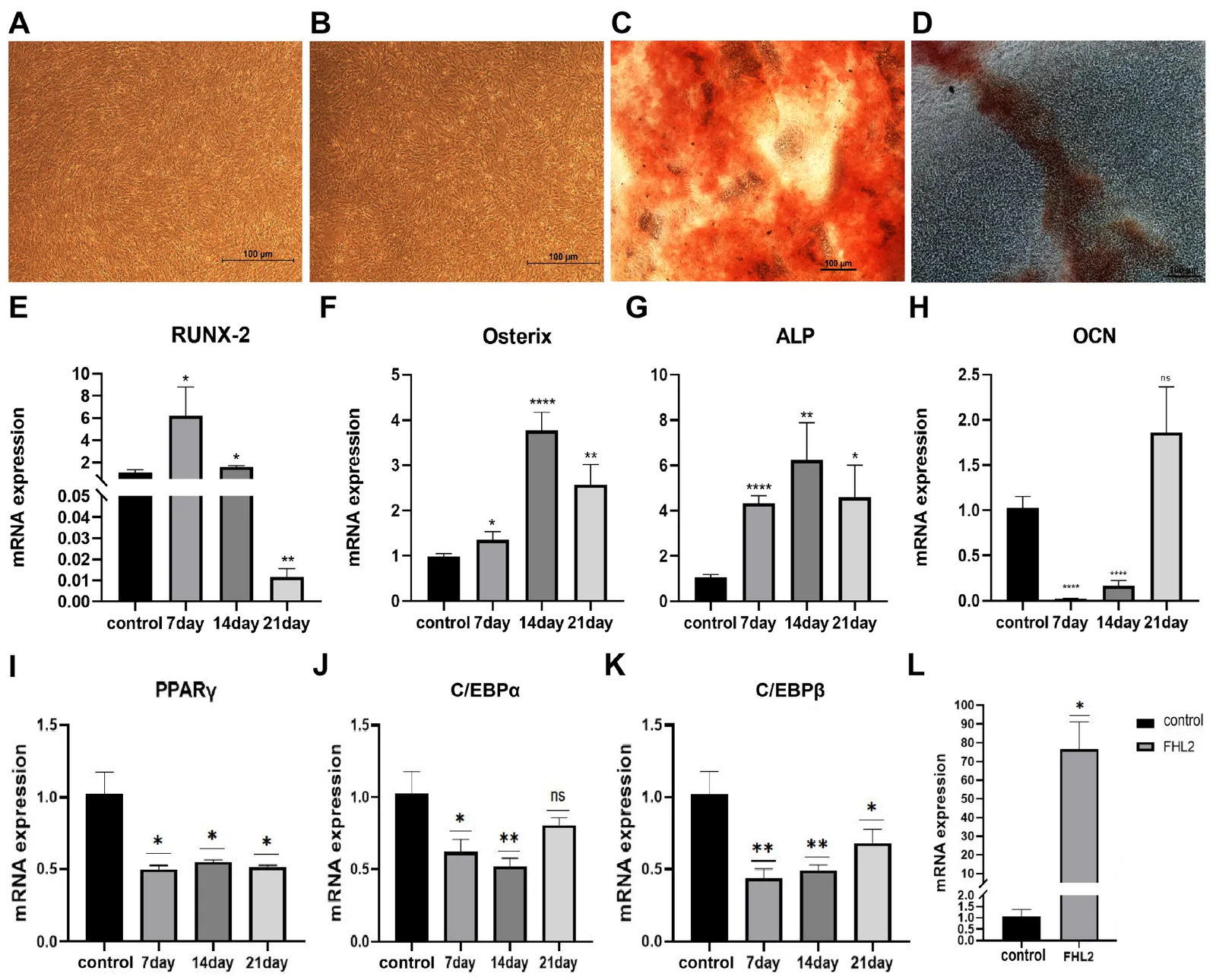
Osteogenic induction of chicken BMSCs and Alizarin Red S staining. (**A**) BMSCs before osteogenic induction. (**B**) BMSCs on day 7 of osteogenic induction. (**C**) Alizarin Red S staining on day 7 of osteogenic induction. (**D**) Representative calcium deposition on day 7. Scale bars = 100 μm. (**E**–**L**) qRT-PCR analysis during osteogenic induction on days 7, 14, and 21: *RUNX2* (**E**), *Osterix* (**F**), *ALP* (**G**), *OCN* (**H**), *PPARγ* (**I**), *C/EBPα* (**J**), *C/EBPβ* (**K**), and *FHL2* (**L**) mRNA expression. For qRT-PCR analysis, data are presented as mean ± SEM from n = 3 independent biological replicates per group; each biological replicate was measured with 3–4 technical replicates. ns, not significant (*p* > 0.05); * *p* < 0.05; ** *p* < 0.01; **** *p* < 0.0001.

During osteogenic induction, control BMSCs showed no obvious morphological changes and no mineralized nodules by Alizarin Red S staining (Figure 2A). In contrast, induced cells progressively exhibited osteogenic-associated morphological remodeling, including a more polygonal morphology, increased cell–cell contacts, multilayer growth, and the appearance of dense clusters. Mineralized nodules became visible with prolonged induction, and Alizarin Red S staining revealed clear calcium deposition in induced cultures (Figure 2B–D). Consistent with these phenotypic changes, qRT-PCR showed time-dependent regulation of osteogenic markers: *RUNX2*, *ALP*, and *Osterix* were increased on day 7, whereas *OCN* was reduced on day 7 but increased over time and was higher on day 21 (Figure 2E–H). In parallel, adipogenic markers (*PPAR*γ, *C/EBPβ*, and *C/EBPα*) were decreased during osteogenic induction (Figure 2I–K). Notably, *FHL2* mRNA expression was increased on day 7 of osteogenic induction (Figure 2L), indicating a positive association between *FHL2* expression and osteogenic differentiation in chicken BMSCs under in vitro induction conditions.

### 3.3. Adipogenic Induction of Chicken BMSCs

**Figure 3:**
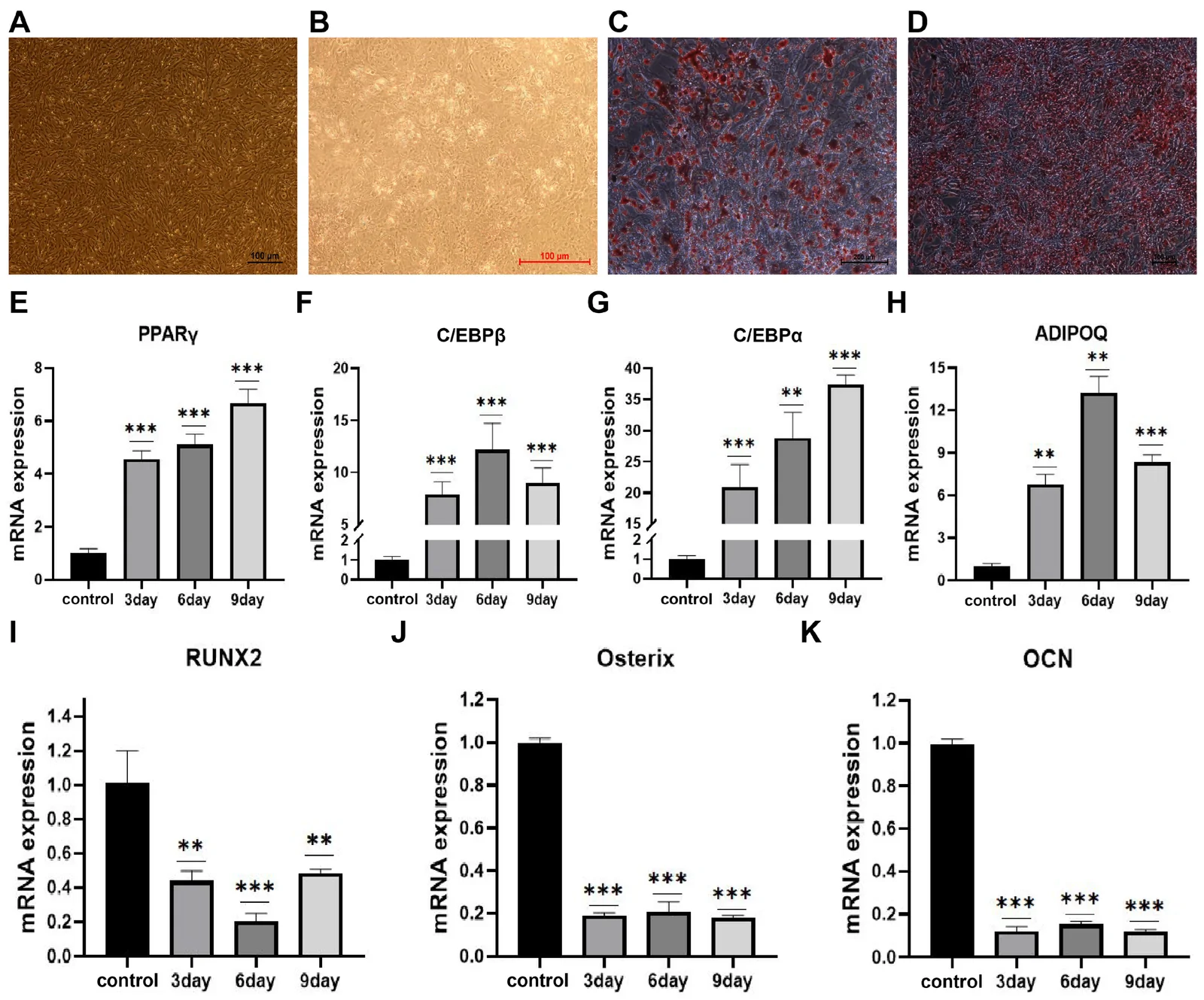
Adipogenic induction of chicken BMSCs and Oil Red O staining. (**A**) BMSCs before adipogenic induction. (**B**) BMSCs on day 6 of adipogenic induction. (**C**,**D**) Oil Red O staining on day 6 of adipogenic induction at two magnifications. Scale bars = 200 μm (**C**) and 100 μm (**D**). (**E**–**K**) qRT-PCR analysis during adipogenic induction on days 3, 6, and 9: *PPARγ* (**E**), *C/EBPβ* (**F**), *C/EBPα* (**G**), *ADIPOQ* (**H**), *RUNX2* (**I**), *Osterix* (**J**), and *OCN* (**K**) mRNA expression. For qRT-PCR analysis, data are presented as mean ± SEM from n = 3 independent biological replicates per group; each biological replicate was measured with 3–4 technical replicates. ** *p* < 0.01; *** *p* < 0.001.

During adipogenic induction, control group BMSCs showed no obvious morphological changes, and Oil Red O staining showed no detectable lipid accumulation (Figure 3A). On day 3, induced cells became more rounded and enlarged, and small translucent lipid droplets appeared in the cytoplasm (Figure 3B). Lipid droplets increased with continued induction. On day 6, Oil Red O staining showed clear red lipid droplets under light microscopy, often with a ring-like appearance (Figure 3C). On day 9, lipid droplet accumulation further increased (Figure 3D). qRT-PCR analysis on days 3, 6, and 9 showed significant induction of adipogenic markers. *PPARγ*, *C/EBPβ*, *C/EBPα*, and *ADIPOQ* were significantly up-regulated on day 3 (*p* < 0.01; Figure 3E–H). *PPARγ* and *C/EBPα* increased further on days 6 and 9 (*p* < 0.01). *C/EBPβ* peaked on day 6 and decreased on day 9 (*p* < 0.001). *ADIPOQ* remained elevated, peaked on day 6, and declined on day 9 (*p* < 0.01). Additionally, osteogenic markers (*RUNX2*, *OCN*, and *Osterix*) were significantly down-regulated during adipogenic induction (*p* < 0.001; Figure 3I–K).

### 3.4. Effects of FHL2 Overexpression and Knockdown on Osteogenic and Adipogenic Markers and Matrix Mineralization

**Figure 4:**
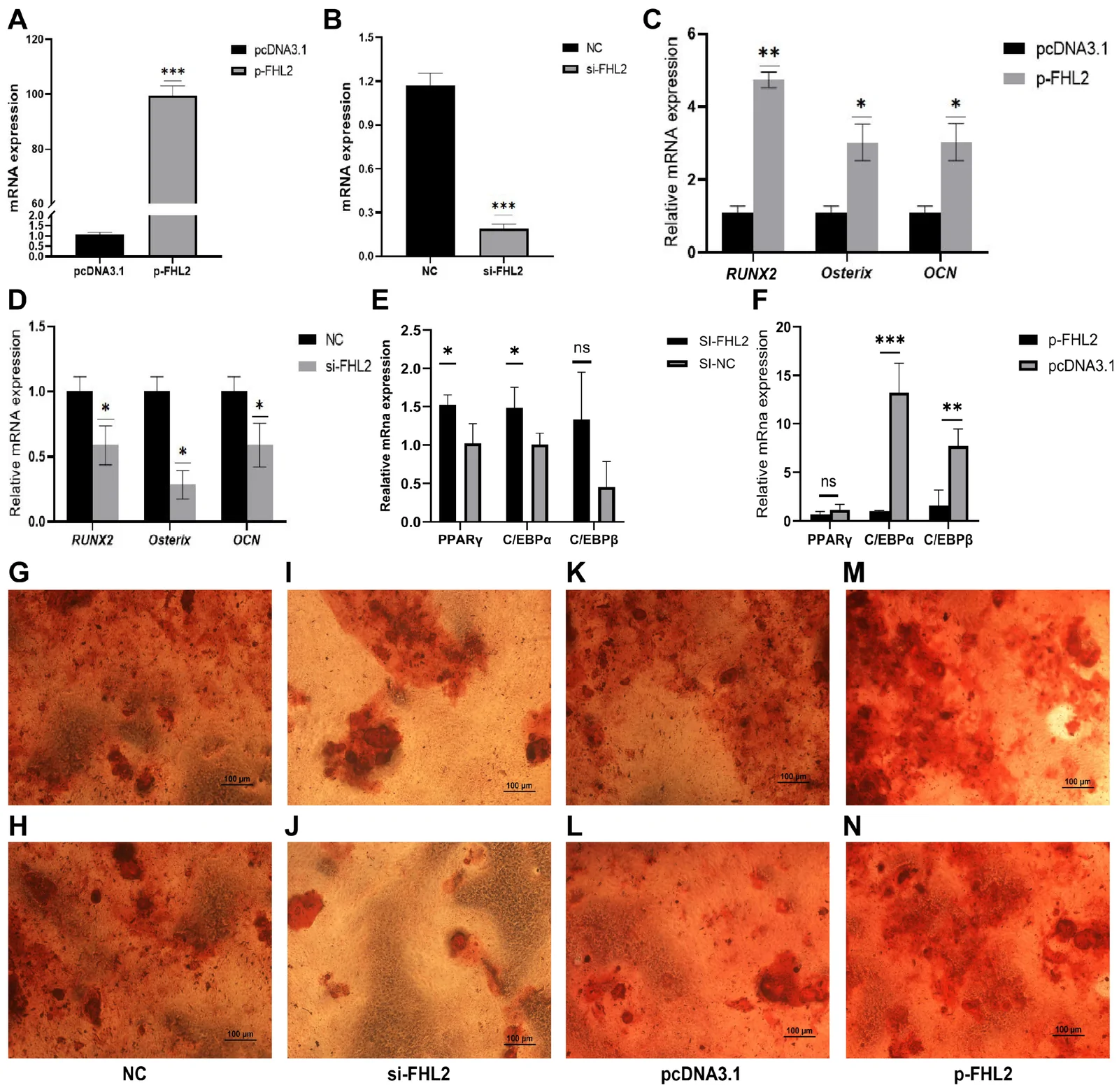
Effects of FHL2 on osteogenic and adipogenic differentiation of chicken BMSCs. (**A**) Transfection efficiency of pcDNA3.1-*FHL2* overexpression in chicken BMSCs. (**B**) Knockdown efficiency of si-*FHL2* in chicken BMSCs. (**C**,**D**) mRNA expression of osteogenic marker genes after *FHL2* overexpression (**C**) or knockdown (**D**). (**E**,**F**) mRNA expression of adipogenic marker genes after *FHL2* knockdown (**E**) or overexpression (**F**). (**G**–**N**) Alizarin Red S staining after osteogenic induction: si-NC control (**G**,**H**), si-*FHL2* (**I**,**J**), pcDNA3.1 control (**K**,**L**), and pcDNA3.1-*FHL2* (**M**,**N**). For qRT-PCR analysis, data are presented as mean ± SEM from n = 3 independent biological replicates per group; each biological replicate was measured with 3–4 technical replicates. Scale bars = 100 μm. ns, not significant (*p* > 0.05); * *p* < 0.05; ** *p* < 0.01; *** *p* < 0.001.

Chicken BMSCs were transfected with an *FHL2* overexpression plasmid (pcDNA3.1-*FHL2*) or *FHL2* siRNA (si-*FHL2*). qRT-PCR confirmed that *FHL2* mRNA levels increased after pcDNA3.1-*FHL2* transfection and decreased after si-*FHL2* transfection (*p* < 0.001; Figure 4A,B). Under these conditions, *FHL2* overexpression increased the mRNA levels of osteogenic markers (*RUNX2*, *Osterix*, and *OCN*), whereas *FHL2* knockdown reduced their expression (*p* < 0.05; Figure 4C,D). In contrast, *FHL2* overexpression significantly reduced the expression of *C/EBPα* and *C/EBPβ*, whereas *PPARγ* did not show a statistically significant change. Conversely, *FHL2* knockdown significantly increased the expression of *C/EBPα* and *PPARγ*, whereas the change in *C/EBPβ* was not statistically significant (*p* < 0.05; Figure 4E,F). Consistent with the gene expression results, Alizarin Red S staining after osteogenic induction showed reduced staining intensity and nodule formation in the FHL2 knockdown group relative to the si-NC control (Figure 4G–J), whereas FHL2 overexpression increased mineralized nodule formation and the Alizarin Red S-positive area relative to the pcDNA3.1 control (Figure 4K–N).

### 3.5. FHL2 Regulates Cell-Cycle Distribution and Proliferation-Related Gene Expression in Chicken BMSCs

**Figure 5:**
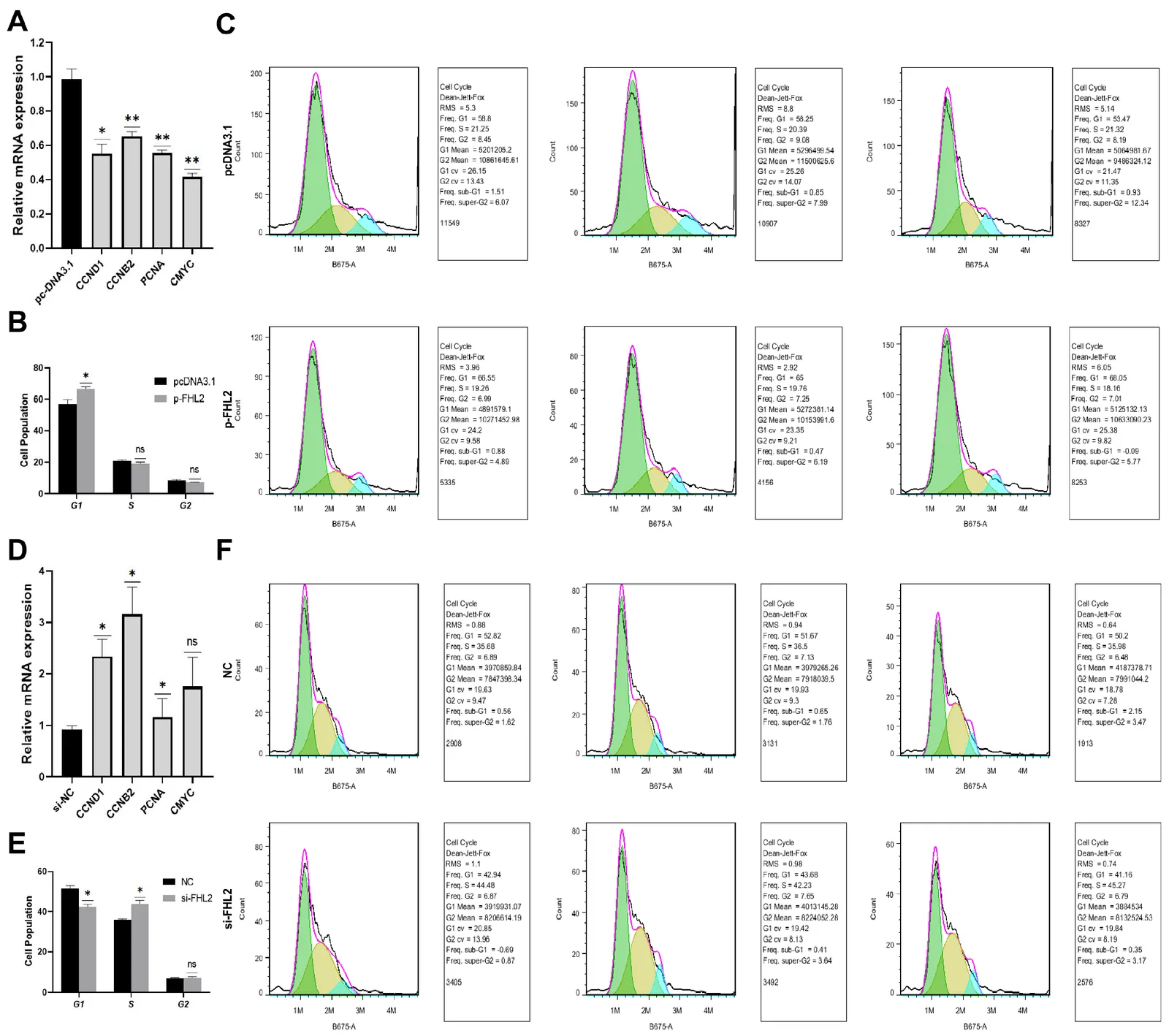
Effects of *FHL2* overexpression and knockdown on proliferation- and cell-cycle-related parameters in chicken BMSCs. (**A**,**D**) qRT-PCR analysis of proliferation- and cell-cycle-related genes (*CCND1*, *CCNB2*, *PCNA*, and *c-MYC*) after *FHL2* overexpression (**A**) or *FHL2* knockdown (**D**). (**C**,**F**) Representative flow-cytometric cell-cycle profiles at 48 h after transfection with pcDNA3.1 or p-*FHL2* (**C**) and si-NC or si-*FHL2* (**F**) (n = 3). (**B**,**E**) Quantification of the percentages of cells in the G1, S, and G2 phases at 48 h after transfection with pcDNA3.1 or p-*FHL2* (**B**) and si-NC or si-*FHL2* (**E**). For qRT-PCR analysis, data are presented as mean ± SEM from n = 3 independent biological replicates per group; each biological replicate was measured with 3–4 technical replicates. ns, not significant (*p* > 0.05); * *p* < 0.05; ** *p* < 0.01.

Chicken BMSCs were transfected with pcDNA3.1-*FHL2* (overexpression) or si-*FHL2* (knockdown), with empty vector (pcDNA3.1) or negative-control siRNA (si-NC) as the corresponding controls. At 48 h post-transfection, qRT-PCR showed that *FHL2* overexpression reduced the mRNA levels of proliferation- and cell-cycle-related genes (*CCND1*, *CCNB2*, *PCNA*, and *c-MYC*; *p* < 0.01; Figure 5A). In contrast, *FHL2* knockdown increased *CCND1*, *CCNB2*, and *PCNA* expression (*p* < 0.05), whereas *c-MYC* showed an increasing trend without reaching statistical significance (Figure 5B). Flow-cytometric analysis further showed that *FHL2* overexpression increased the proportion of cells in G1 phase (*p* < 0.05; Figure 5C,D), while the S- and G2-phase fractions did not differ significantly from controls. Conversely, *FHL2* knockdown increased the S-phase fraction and reduced the G1-phase fraction (both *p* < 0.05; Figure 5E,F), with no significant change in G2 phase. Together, these data support an association between *FHL2* expression and altered cell-cycle distribution in chicken BMSCs.

### 3.6. FHL2 Suppresses Apoptosis in Chicken BMSCs

**Figure 6:**
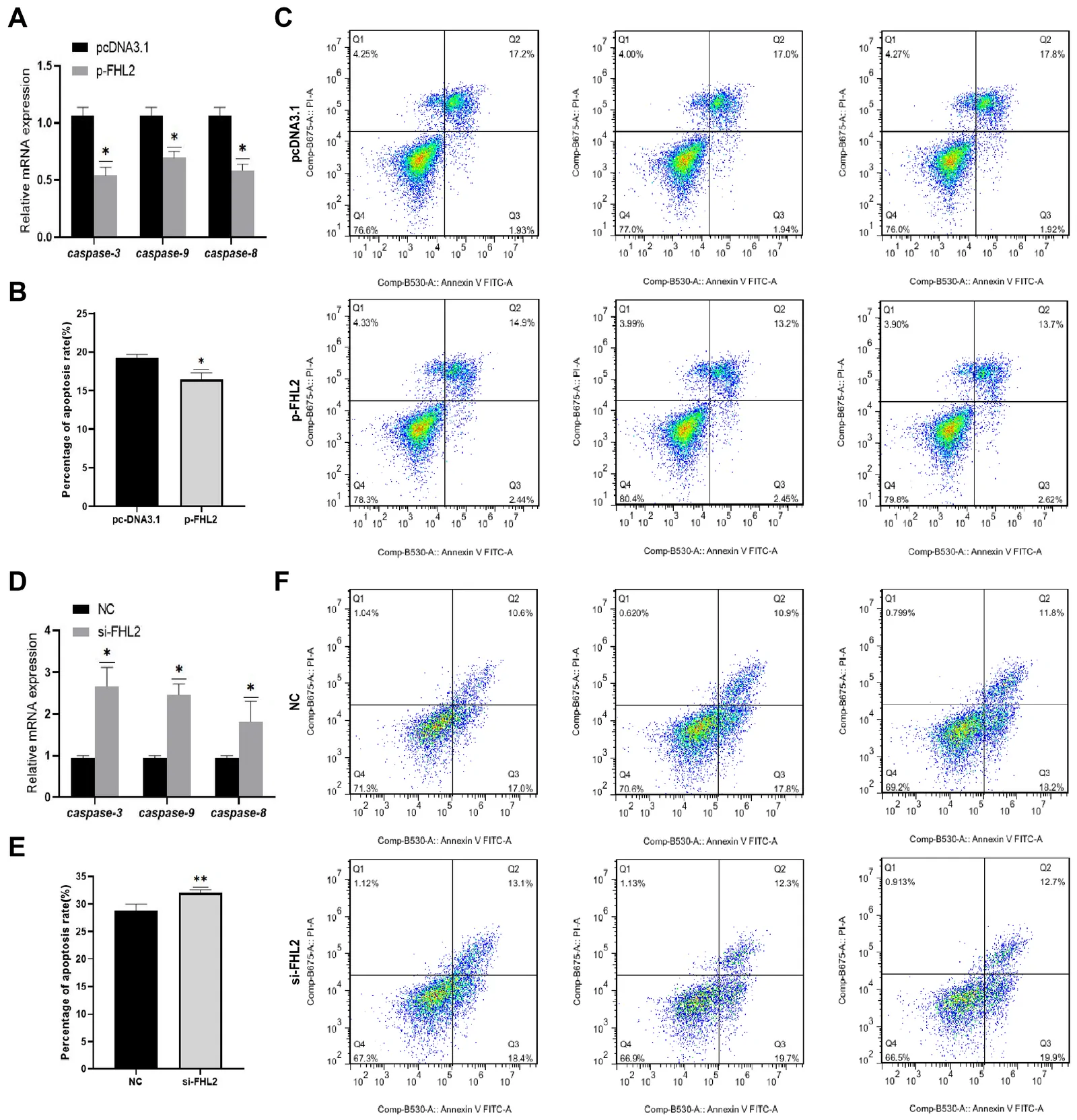
Effects of *FHL2* overexpression and knockdown on apoptosis in chicken BMSCs. (**A**,**D**) RT-qPCR analysis of apoptosis-related genes (*Caspase-3*, *Caspase-9*, and *Caspase-8*) after *FHL2* overexpression (**A**) or *FHL2* knockdown (**D**). (**C**,**F**) Representative flow-cytometric apoptosis profiles at 48 h after transfection with pcDNA3.1 or p-*FHL2* (**C**) and si-NC or si-*FHL2* (**F**) (n = 3). (**B**,**E**) Quantification of the apoptosis rate at 48 h after transfection with pcDNA3.1 or p-*FHL2* (**B**) and si-NC or si-*FHL2* (**E**). For qRT-PCR analysis, data are presented as mean ± SEM from n = 3 independent biological replicates per group; each biological replicate was measured with 3–4 technical replicates. * *p* < 0.05; ** *p* < 0.01.

Chicken BMSCs were transfected with pcDNA3.1-*FHL2*. At 48 h post-transfection, qRT-PCR showed that *FHL2* overexpression reduced the mRNA levels of apoptosis-related genes (*Caspase-3*, *Caspase-8*, and *Caspase-9*; *p* < 0.05; Figure 6A), whereas *FHL2* knockdown increased their expression (*p* < 0.05; Figure 6B). Flow-cytometric analysis further showed that *FHL2* overexpression increased the viable cell fraction and reduced the apoptotic rate compared with the control group (*p* < 0.05; Figure 6C,D). Conversely, *FHL2* knockdown decreased the viable cell fraction and increased the apoptotic rate (*p* < 0.01; Figure 6E,F).

### 3.7. RNA-Seq Analysis After FHL2 Knockdown in Chicken BMSCs

**Figure 7:**
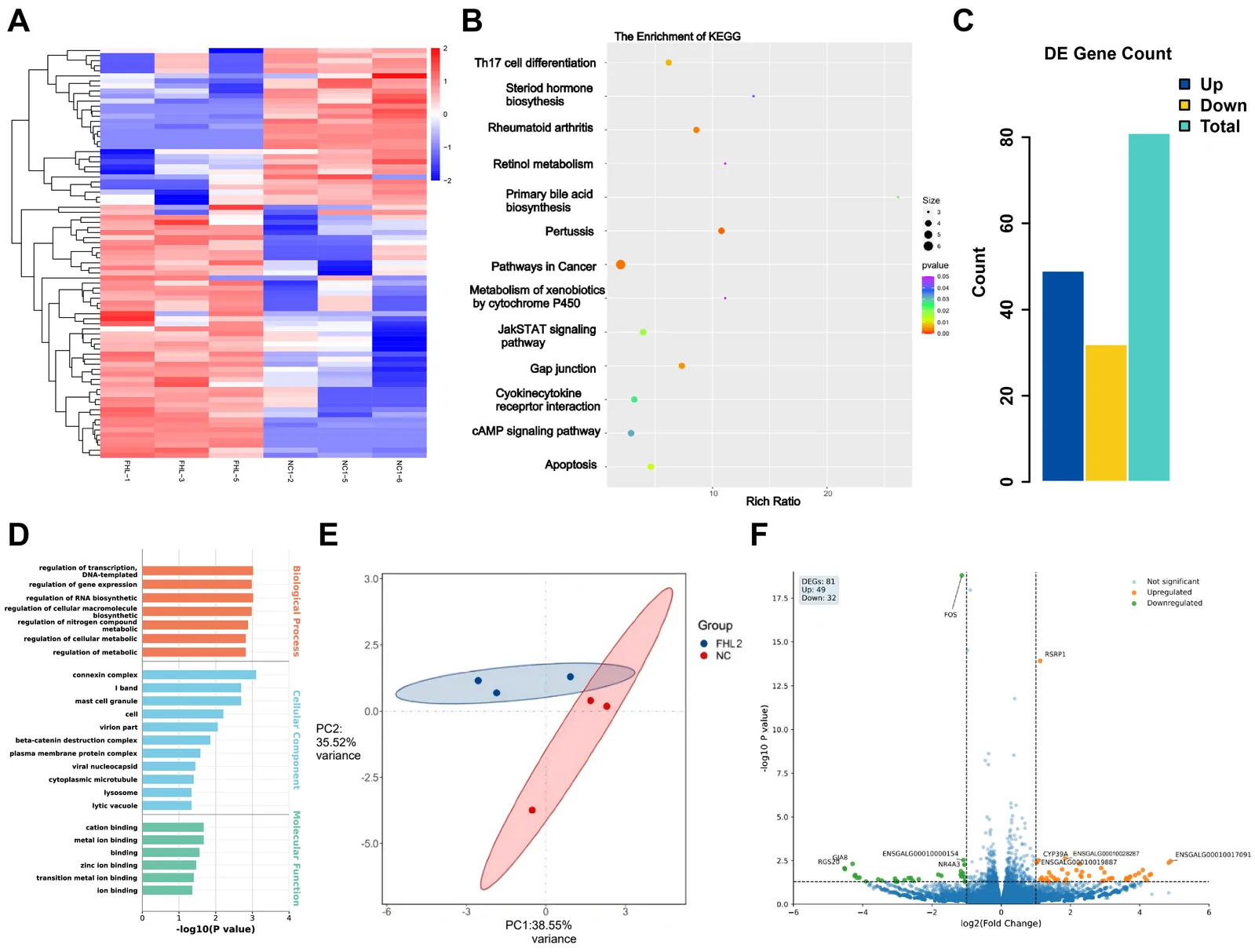
Transcriptomic profiling and functional enrichment analysis of chicken BMSCs in the si-FHL2 and si-NC groups. (**A**) Hierarchical clustering heatmap of differentially expressed genes. (**B**) Kyoto Encyclopedia of Genes and Genomes pathway enrichment analysis of differentially expressed genes. (**C**) Summary of the numbers of up-regulated, down-regulated, and total differentially expressed genes between the two groups. (**D**) Gene Ontology enrichment analysis of differentially expressed genes, including biological process, cellular component, and molecular function categories. (**E**) Principal component analysis of the transcriptomic profiles of the si-FHL2 and si-NC groups. (**F**) Volcano plot of differentially expressed genes.

Principal component analysis based on the gene count matrix showed clear separation between the si-FHL2 and si-NC groups (Figure 7E). Differential expression analysis was performed using the limma-voom pipeline. Genes with |log2(fold change)| ≥ 1 and an unadjusted *p* value < 0.05 were defined as differentially expressed. In total, 81 differentially expressed genes were identified, including 49 up-regulated and 32 down-regulated genes (Figure 7C).

A volcano plot showed the distribution of up-regulated and down-regulated DEGs between the si-FHL2 and si-NC groups (Figure 7F). Hierarchical clustering of these DEGs clearly separated the two groups (Figure 7A). GO enrichment analysis showed that these DEGs were primarily involved in the regulation of transcription, gene expression, RNA biosynthetic processes, cellular metabolic processes, and ion binding (Figure 7D). KEGG pathway analysis further identified significant enrichment in pathways associated with signal transduction, apoptosis, immune-related responses, and metabolic regulation, including the JAK–STAT signaling pathway, cAMP signaling pathway, steroid hormone biosynthesis, and retinol metabolism (Figure 7B).

### 3.8. Validation of RNA-Seq Differential Expression Trend

**Figure 8:**
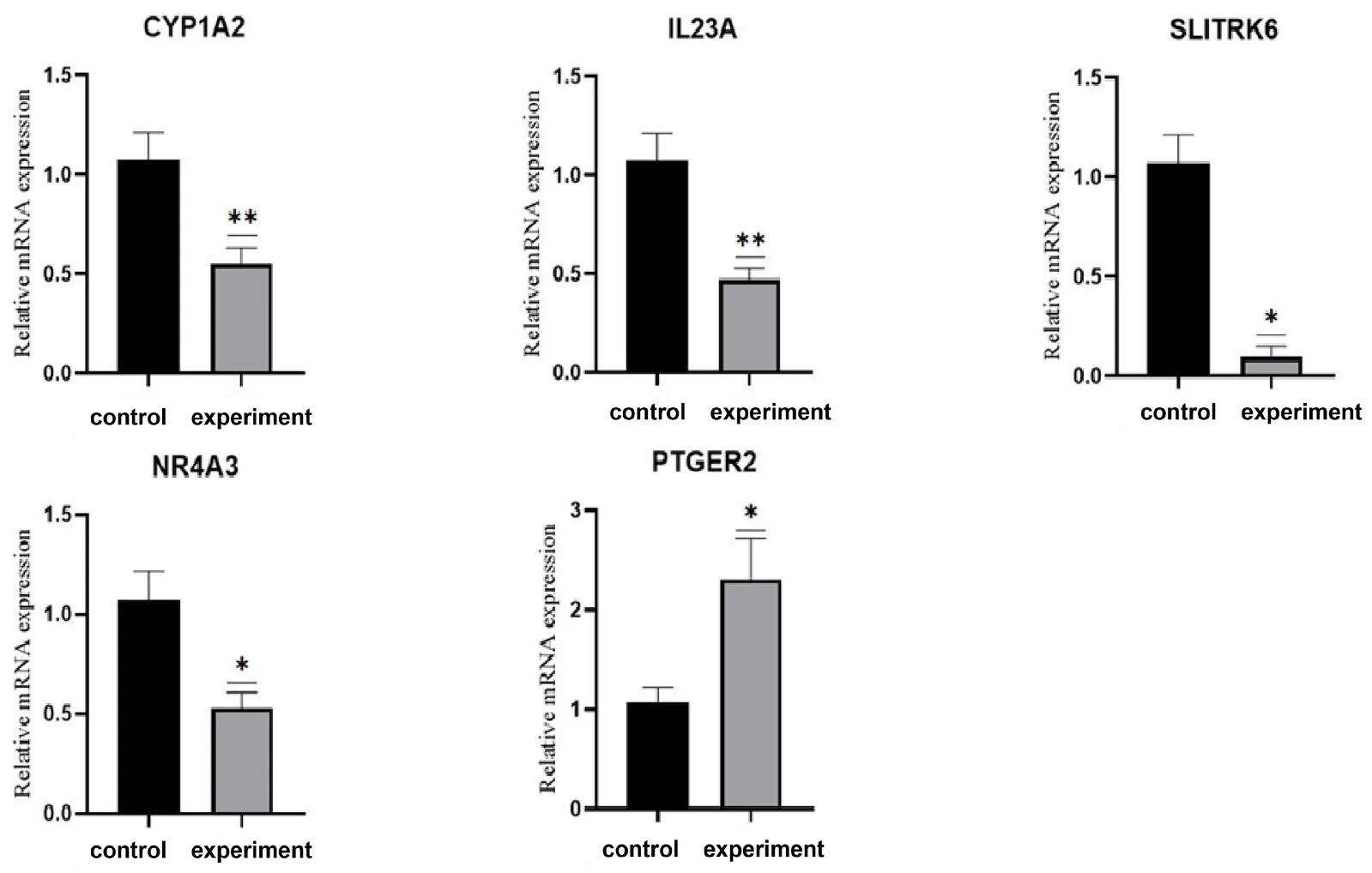
qRT-PCR validation of differentially expressed genes identified by RNA-seq. Relative mRNA expression of selected differentially expressed genes in chicken BMSCs after *FHL2* knockdown, including *CYP1A2*, *IL23A*, *SLITRK6*, *NR4A3*, and *PTGER2*. For qRT-PCR analysis, data are presented as mean ± SEM from n = 3 independent biological replicates per group; each biological replicate was measured with 3–4 technical replicates. * *p* < 0.05; ** *p* < 0.01.

To validate the RNA-seq data, we selected five key differentially expressed genes from the si-*FHL2* transcriptome. These genes are biologically connected to the enriched KEGG pathways. Four genes were down-regulated (*CYP1A2*, *IL23A*, *SLITRK6*, and *NR4A3*). One gene was up-regulated (*PTGER2*). We measured their mRNA expression levels by qRT-PCR in chicken BMSCs after *FHL2* knockdown. *CYP1A2*, *IL23A*, *SLITRK6*, and *NR4A3* were significantly down-regulated. *PTGER2* was significantly up-regulated (Figure 8). The trends matched the RNA-seq results. This supports the reliability of the transcriptomic analysis.

## 4. Discussion

### 4.1. Osteogenic Marker Dynamics During Induction

Osteogenic differentiation is regulated by multiple transcription factors and signaling pathways and is central to skeletal development and regeneration [20]. Osteogenic markers, including RUNX2, ALP, Osterix, and OCN, typically exhibit stage-dependent expression patterns that reflect early differentiation, matrix maturation, and late mineralization [21]. In chicken BMSCs specifically, dynamic transcriptome analysis has shown coordinated gene-expression changes during osteogenic differentiation, providing species-specific support for interpreting the marker changes observed in the present study [22]. Transcriptional dynamics were accompanied by progressive mineralized nodule formation and calcium deposition detected by Alizarin Red S staining, indicating successful osteogenic induction in chicken BMSCs and providing a basis for interpreting downstream regulatory mechanisms.

### 4.2. FHL2 Promotes Osteogenic Differentiation and Inhibits Adipogenic Differentiation

A major finding of the present study was the early upregulation of *FHL2* on day 7 of osteogenic induction, indicating that FHL2 acts at an early stage of osteogenic differentiation. Adipogenesis-related transcripts decreased with *FHL2* overexpression and increased after *FHL2* knockdown, indicating that FHL2 is consistently linked to a shift toward osteogenic rather than adipogenic differentiation in chicken BMSCs. This interpretation is consistent with evidence that adipogenic differentiation in chicken BMSCs is regulated by growth and metabolic signaling; for example, GHR suppresses adipogenic differentiation by affecting mitochondrial biogenesis and mitochondrial function [23].

In addition, previous studies have reported that FHL2 acts as a coactivator of RUNX2 and enhances its transcriptional activity. For example, in dental pulp stem cells, FHL2 binds to RUNX2 and increases the expression of osteogenic genes, including alkaline phosphatase and type I collagen. During mouse tooth development, FHL2 and RUNX2 also exhibit similar embryonic expression patterns and distinct subcellular localization, indicating coordinated roles in developmental regulation [24]. Based on the above experimental results, FHL2 promotes osteogenic transcriptional differentiation while inhibiting adipogenic differentiation. However, direct pathway-level validation will be necessary to define the causal mechanism in chicken BMSCs. Thus, the present results should be viewed as consistent with, but not direct proof of, the FHL2-p53/β-catenin mechanism reported during MSC osteogenic differentiation [9].

### 4.3. FHL2 Regulates Proliferation, Cell-Cycle Progression, and Apoptosis in Chicken BMSCs

Differentiation of mesenchymal progenitors is tightly associated with cell cycle and survival, and changes in proliferation or apoptosis can alter both the number of available progenitors and the distribution of differentiation-competent subpopulations. Our results revealed that FHL2 affects the proliferative capacity and cell-cycle progression of chicken BMSCs. These observations indicate that elevated FHL2 is associated with reduced proliferative activity in chicken BMSCs under our experimental conditions, which may contribute to cell differentiation by altering the timing of cell-cycle exit or the differentiation window.

A similar trend was observed for apoptosis-related outcomes, with FHL2 exerting a protective effect against BMSC apoptosis. These results indicate that FHL2 is associated with enhanced survival of chicken BMSCs in vitro. Importantly, published data indicate that FHL2 can exert divergent effects across cellular contexts. In osteosarcoma U2OS cells, *FHL2* knockdown inhibited proliferation, promoted apoptosis, and induced G0/G1 arrest, indicating a role in cell-cycle control and tumor cell survival [25]. Other studies have also indicated that FHL2 can promote proliferation and migration under certain conditions, consistent with proposed oncogenic functions [26]. These differences indicate that FHL2-dependent regulation of proliferation and apoptosis is likely microenvironment-dependent and that the direction of its effects may depend on signaling states, differentiation stage, and cell identity. In chicken BMSCs, our data support the view that FHL2-associated changes in proliferation and apoptosis accompany shifts in osteogenic and adipogenic transcriptional programs.

### 4.4. Transcriptomic Signatures After FHL2 Knockdown Indicate Multiple Downstream Programs

To further investigate the role of the *FHL2* gene in the osteogenic–adipogenic differentiation balance of chicken BMSCs, we analyzed transcriptome sequencing data after *FHL2* knockdown. Differentially expressed genes and enriched pathways were associated with immune and inflammatory signaling, metabolic regulation, transcriptional control, and cytoskeletal organization. At the individual-gene level, some DEGs, such as *SLITRK6*, have been annotated in processes related to nervous system development, whereas *NR4A3* has been associated with atherosclerosis and inflammatory responses [27,28]. *CYP1A1*, *CYP1A2*, and *CYP39A1* are implicated in xenobiotic/drug metabolism and have also been linked to lipid synthesis and immune regulation. *RGS20* modulates downstream G protein signaling, including Gαo-mediated cAMP signaling [29]. *TUBAL3* was also identified among the genes associated with microtubule-based processes and cytoskeletal organization in our transcriptomic analysis. Inflammatory and immune-related genes, including *IL23A* and *PTGER2*, were also identified among the differentially expressed genes in our transcriptomic analysis. These findings suggest a possible involvement of immune and inflammatory signaling; however, the specific functions of these genes were not directly validated in the present study. Rather than assigning direct osteogenic/adipogenic functions to each gene, these enrichments collectively indicate that *FHL2* knockdown leads to changes in signaling, immune, and metabolic networks, as well as cytoskeletal dynamics, which may indirectly influence cell differentiation in BMSCs.

KEGG enrichment further highlighted pathways related to Th17 cell differentiation and apoptosis, as well as JAK–STAT and cAMP signaling, steroid hormone biosynthesis, and retinol metabolism. The JAK–STAT pathway regulates proliferation, differentiation, apoptosis, and immune responses, and FHL2 has been proposed to act as a signaling adaptor in pathways including MAPK and JAK–STAT, potentially bridging signaling components and facilitating signal transmission [30]. A major downstream effector of cAMP signaling is CREB; calcium and cAMP signals can converge on CREB through different routes, producing long-term transcriptional changes [31]. cAMP-dependent cascades can regulate *CYP19A1* transcription; *CYP19A1* encodes steroid aromatase (P450 19A1), a cytochrome P450 enzyme that converts androgens to estrogens and is expressed in tissues such as adipose tissue, brain, ovary, and placenta [32]. In ovarian granulosa cells, FHL2 interacts with NR5A family nuclear receptors and CREB, and enhances NR5A—dependent transcriptional activation of the INHA promoter under cAMP stimulation [4]. In summary, these observations are consistent with the enrichment of cAMP-related signaling and steroid/retinoid metabolic pathways after *FHL2* knockdown and support a working model in which FHL2 contributes to osteogenic–adipogenic balance through coordinated modulation of differentiation/apoptosis pathways as well as broader signaling and metabolic networks.

### 4.5. Limitations and Future Directions

An important limitation of this study is that the characterization of the isolated cells was based primarily on immunofluorescence staining using a limited marker panel. Although CD29, CD105, and CD166 showed positive signals and no detectable CD45 signal was observed, immunofluorescence staining alone cannot quantitatively establish the purity or homogeneity of the isolated cell population. Although independent cell batches were used for cell characterization and the functional assays, a quantitative purity assessment of the matched functional batches was not performed. The cells should therefore be interpreted as a chicken bone marrow-derived stromal/MSC-like population. Future studies should apply a unified, chicken-validated marker panel together with quantitative flow-cytometric analysis to characterize matched cell batches before functional experiments.

This study was conducted primarily in vitro using chicken BMSCs, and most mechanistic inferences were based on transcriptional data and pathway enrichment analysis. Future work should validate key pathway nodes at both the protein and functional levels, for example by assessing Wnt/β-catenin activity and determining whether FHL2-dependent regulation of bone–lipid balance can be reproduced in vivo or in relevant breeding populations. In addition, targeted validation of top differentially expressed genes from RNA-seq and integration with functional assays will further refine the downstream regulatory network controlled by FHL2.

## 5. Conclusions

Overall, our results indicate that FHL2 is involved in the osteogenic–adipogenic differentiation balance of chicken BMSCs. *FHL2* overexpression increased osteogenic marker expression and matrix mineralization while reducing adipogenesis-related transcripts. In contrast, *FHL2* knockdown produced the opposite results. Manipulation of FHL2 also affected proliferation-related gene expression and cell-cycle distribution, and it was accompanied by changes in apoptosis-related parameters in vitro. RNA-seq analysis after *FHL2* knockdown pointed to pathways linked to lipid-associated processes, immune/inflammatory regulation, cytoskeletal organization, and signal transduction. JAK–STAT and cAMP signal pathways were enriched. Pathway analysis also raised the possibility that Wnt/β-catenin signaling may be involved, although direct validation is still required. In summary, FHL2 may influence the differentiation of BMSCs through several pathways, and these findings provide candidate molecular evidence for future studies on skeletal development and fat deposition in chickens, but in vivo validation is still required.

## Data Availability

The RNA-seq data supporting the findings of this study have been deposited in OMIX (Open Archive for Miscellaneous Data), National Genomics Data Center (NGDC), China National Center for Bioinformation, under accession number OMIX015289, and are publicly available. The data are publicly available at: https://ngdc.cncb.ac.cn/omix/release/OMIX015289 (accessed on 7 October 2026).

## Supplementary Materials

The following supporting information can be downloaded at https://www.mdpi.com/article/10.3390/ani16203197/s1. Table S1: Gene primer table; Supplementary Material File S2: Information on siRNA and plasmid synthesis.
