# Supplementary material for: Study on the Role of FHL2 in Chicken: Promoting Bone Growth and Inhibiting Fat Formation via Regulating BMSC Differentiation Balance
Source: Animals (Basel). 2026 Oct 12;16(20):3197. doi: 10.3390/ani16203197 (PMC13700831; doi:10.3390/ani16203197)
Supplement: Supplementary file 1 [file animals-16-03197-s001.zip › Supplementary Material File S2/COA of FHL2.pdf]

SERVICE TYPE: DNA CONSTRUCT

Certificate of Analysis

Project ID: GS1-24040290

Construct Information

**Gene Name**	FHL2	**Gene Length**	852 bp
**Cloning Vector**	pcDNA3.1(+)	**Cloning Strategy**	NheI XhoI
**Construct Resistant**	Amp	**Construct ID**	S03248-140851

QC Items

QC Items	Method	Specifications	Results
Target Sequence	Sequence alignment	Sequencing results are consistent to the confirmed sequence.	Pass
Vector Sequence	Sequence alignment	20bp flanking sequences of the vector are correct.	NA
Reading Frame	Sequence alignment	Frame is correct and consistent to the client's requirement.	Pass
Fragment Size	Restriction Digests	The size of inserted fragment is right and free of any contaminated bands.	Pass
Quality/Quantity	UV spectrophotometry	Miniprep: OD260/280=1.7~2.0<br>4μg/tube, 1 tube, Lyophilized	Pass
Appearance	Visual inspection	Clear and free of foreign particles.	Pass
Customized Test	NA	NA	NA

Restriction Digestion Map

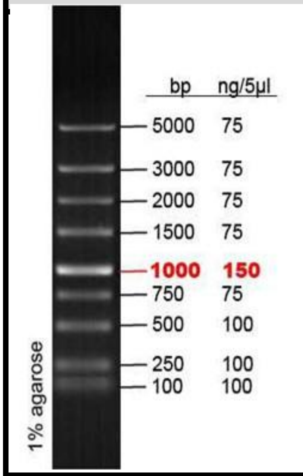

M 1 2

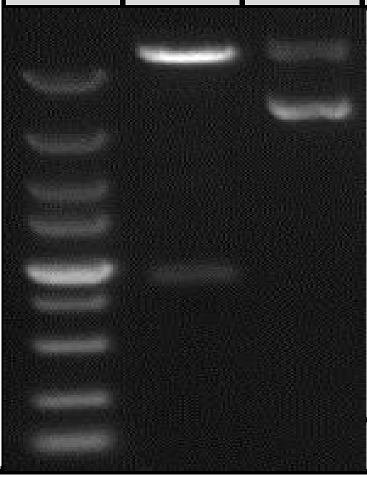

Lane M: DNA Marker

Lane 1: Plasmid digested by NheI XhoI

Lane 2: Plasmid DNA

Date: 2024-4-24
