# Supplementary material for: Study on the Role of FHL2 in Chicken: Promoting Bone Growth and Inhibiting Fat Formation via Regulating BMSC Differentiation Balance
Source: Animals (Basel). 2026 Oct 12;16(20):3197. doi: 10.3390/ani16203197 (PMC13700831; doi:10.3390/ani16203197)
Supplement: Supplementary file 1 [file animals-16-03197-s001.zip › Supplementary Material File S2/Si-Rna FHL2.pdf]

The siRNA sequence information for the chicken *FHL2* gene is as follows:

Gene name	Sequence (5′ →3′)
FHL2-1	CCAAGTACATCTCATTCGA
FHL2-2	CAACCAATTGGGACAAAGA
FHL2-3	CTGATTGCTACTCCAATGA

The sequence 2 highlighted in yellow is the final siRNA chosen.

Position:

atgac tgagcgtttc gactgccact actgcaaaga gtccctgttt ggtaagaagt
121 acatcctgag ggaggacagc ccctactgcg tgaaatgcta tgaaaacctc tattccaaca
181 cctgtgagga atgcaagaaa cctattggag ctgactgcaa ggatctgtct tacaaggacc
241 gccactggca tgaaacctgc ttccactgct tccagtgcaa gaattcattg gtggacaagc
301 cttttgctgc aaaagaggag catctacttt gtactgattg ctactccaat gaatactctt
361 ccaaatgtaa tgagtgcaag aagactatta tgccaggtac tcggaagatg gaatacaaag
421 gcaacagctg gcacgagacc tgctttatct gctaccgctg ccagcaacca attgggacaa
481 agagtttcat tcctaaagac aatcaaaatt tttgtgttcc ctgctatgaa aagcagtttg
541 ccatgcagtg cgtccagtgc aagaaggcta tcactacagg aggtgtgact taccgggagc
601 agccttggca taaggagtgc tttgtctgta ctggatgcaa gaagcagttg tctggacaac
661 gctttacctc ccgggatgag tttgcatatt gcctgagttg cttctgcaat ctctatgcca
721 aaaagtgtgc tggatgcaca aacccaatca gtggtctcgg aggaaccaag tacatctcat
781 tcgaagagcg gcagtggcac aatgattgct ttaattgtaa gaagtgttct ctctcattag
841 taggccgtgg cttccttaca gaaagggatg acatcctctg ccctgaatgt ggaaaggata
901 tctaa
