# Supplementary material for: Study on the Role of FHL2 in Chicken: Promoting Bone Growth and Inhibiting Fat Formation via Regulating BMSC Differentiation Balance
Source: Animals (Basel). 2026 Oct 12;16(20):3197. doi: 10.3390/ani16203197 (PMC13700831; doi:10.3390/ani16203197)
Supplement: Supplementary file 1 [file animals-16-03197-s001.zip › Table S1-Gene primer table.pdf]

Gene name	Sequence (5′ →3′)	Annealing temperature（℃）
RUNX2	F-CCACAAGGACAGAGTCAGATTACAG	58
	R-GGAGTGAATGGACGGCGAAGT	
ALP	F-TGCACACCAGCTTTCCTTGA	58
	R-GCCAGATGAGTACAGCCCAA	
Osterix	F-CGTTCGTCTGCAATTGGCTCTTCT	58
	R-GGTGTTTGCTGAGGTGGTCGGA	
OCN	F-GCGGCCGTCTCGCAAAG	57
	R-GCAGCGACTGCGGTTTGG	
PPARγ	F-CCAGCGACATCGACCAGTTA	56
	R-AGAGCGAAACTGACATCGCT	
C/EBPα	F-TTCTACGAGGTCGATTCCCG	56
	R-GAGTGCTCGTTCTCGCAGAT	
C/EBPβ	F-TCCTTTCCGATCTCTTCGCC	56
	R-GGCTCCACTTTGGTCTCCAC	
C-MYC	F-AAAGAGCAGTTGAGGCGGAG	56
	R-AGATTCGGCCAAGGTTGTGT	
PCNA	F-AATGCGGATACGTTGGCTCT	56
	R-CACCAATGTGGCTGAGGTCT	
CCND1	F-TGCGGTCACCCCTCAAA	56
	R-GTCTGATGGAGTTGTCGGTGT	
CCNB2	F- CGAGGCGTGTTTGTGTAGAG	60
	R-TCCAAAACAGCCCTCTTGCC	
Caspase-3	F-GTGGTGGAGGTGGAGGAG	60
	R-ATCTTCCCCTGAGCGTGGT	
Caspase-8	F-TATGAAGTACAGTGCCGCCC	60
	R-AGACGGGAGCTTTCTGACAC	
Caspase-9	F-GGAATGAGGACGAGCCAGAC	60
	R-TGTACCTCCCCTTGGTGGAT	
CYP1A2	F-GGAGCCTCATGTACCTCGTG	60
	R-CCGATGGTCTGATCCAGCTC	
IL23A	F-GAACAGCACGCTCTGCCT	60
	R-GTGTGGCGAAGATGTCGGAG	
SLITRK6	F-AGCACAGAGAACACAGAAGCA	58
	R-ACAAAGCGTCCAATCGATCAC	
NR4A3	F-ATTCAGCGCGCAAGATATGC	60
	R-CTTGCCATAGTCCGGGTTCA	
PTGER2	F-TCCCTGCCCTTCACGATCT	60
	R-GGGTTGATGGAGAGGAAGCG	
GAPDH	F: CGTCTGGAGAAACCAGCCAA	60
	R: TCCTTGGATGCCATGTGGAC	
